# Frequency and spectrum of Wolcott–Rallison syndrome in Saudi Arabia: a systematic review

**DOI:** 10.3402/ljm.v8i0.21137

**Published:** 2013-06-10

**Authors:** Abdelhadi M. Habeb

**Affiliations:** Endocrine and Diabetes Unit, Maternity and Children Hospital, Al-Madinah, Saudi Arabia

**Keywords:** Wolcott–Rallison syndrome, *EIF2AK3* mutations, Saudi Arabia, neonatal diabetes

## Abstract

**Background:**

Wolcott–Rallison syndrome (WRS) is caused by recessive *EIF2AK3* gene mutations and characterized by permanent neonatal diabetes (PNDM), skeletal dysplasia, and recurrent hepatitis. The frequency of this rare syndrome is largely unknown.

**Objectives:**

To define the frequency and spectrum of WRS in the Kingdom of Saudi Arabia (KSA) based on published data.

**Methods:**

The Medline database was searched for published articles on WRS. The number of reported cases from KSA was compared to the total number of WRS cases reported worldwide. The genotype and phenotype of WRS patients from KSA were reviewed.

**Results:**

Ten articles describing 23 WRS patients from 12 Saudi families from 1995 to 2012 were identified. This figure accounts for 27.7% (23/83) of the patients and 22.2% (12/54) of the families with WRS reported worldwide until January 2013. All Saudi patients with WRS presented with PNDM, and they represent 59% of all PNDM cases from WRS. At reporting, 73% of patients experienced recurrent hepatitis, 56.5% had skeletal abnormalities, and 39.1% of them were dead. There was a variation in the phenotype even between affected siblings. Genetic diagnosis was confirmed in all 12 families with no correlation between the genotype and phenotype. Eight of the nine *EIF2AK3* mutations were only reported in these families, and one was shared with a patient from Qatar, a neighboring Arab state.

**Conclusions:**

No study on the frequency of WRS has been published. However, the available data indicate that KSA has the largest collection of patients with WRS worldwide, and nine of the identifiable *EIF2AK3* mutations appear to be confined to Arabs. Establishing a national or international registry for WRS would provide more reliable data on this rare condition.

Wolcott–Rallison syndrome (WRS) is an autosomal recessive condition that was first described in three children in 1972 (1). It is characterized by permanent neonatal diabetes mellitus (PNDM), skeletal dysplasia, and recurrent hepatitis triggered by viral illnesses and stress (2). Other features such as renal dysfunction, failure to thrive, neutropenia, exocrine pancreatic insufficiency, hypothyroidism, recurrent infection, and developmental delay have been reported in some patients (3–5). The condition has poor prognosis, with most patients dying during childhood mainly due to acute fulminant hepatitis and/or acute renal impairment (2–5).

In 2000, mutations in the *EIF2AK3* gene were identified in a few patients with WRS (5), and since then almost all patients with classical WRS features have *EIF2AK3* mutations. The *EIF2AK3* gene encodes a transmembrane protein called protein kinase R-like endoplasmic reticulum kinase (PERK), which is important for the cellular response to endoplasmic reticulum (ER) stress. The absence of PERK activity reduces the ER's abilities to deal with stress, leading to cell death by apoptosis in many tissues (6, 7). Recently, reports by Feng et al. (8) and Gupta et al. (9) showed that acute ablation of PERK results in reduced cell proliferation with abnormal insulin trafficking and secretion. Studies in EIF2AK3/PERK knockout mouse demonstrated that PERK is required for pancreatic beta cell development during fetal and early neonatal development (7), and that diabetes and skeletal dysplasia are due to loss of PERK expression in pancreatic beta cells (10) and osteoblasts (11).

Data on the epidemiology of WRS are limited, and the latest literature review on the subject suggested that less than 60 WRS cases were reported worldwide (2). However, the condition has been recently found to be the commonest genetic cause of PNDM in consanguineous families (4) and in the Arab population (12). In the Kingdom of Saudi Arabia (KSA), the population is mostly Arab, and consanguinity is highly practiced (13); it is therefore expected to be one of the demographic foci for this condition. However, little is known about the frequency or spectrum of WRS in the country. The aim of this article is therefore to define the frequency of WRS in KSA compared to other populations and to describe the genetics and clinical phenotype of WRS in the country based on reported cases in the literature.

## Methods

### Article collection

The Medline database was searched for articles, including case reports and letters, using the key words ‘Wolcott–Rallison syndrome’, ‘permanent neonatal diabetes’, and ‘*EIF2AK3* gene mutations’. The titles and abstracts of the papers were scanned by the author to identify eligible articles according to defined inclusion and exclusion criteria. The references in selected papers were checked for additional articles. Replication of reported cases was excluded by patients’ demographic data and clinical characteristics, the mutation type, and, if needed, authors’ names and institutions.

### Inclusion and exclusion criteria

Articles were selected if they were written in English, were published before 31 January 2013, and included data on the epidemiology, phenotype, or genotype of WRS subjects of Saudi origin. Studies including Saudi patients with PNDM not caused by WRS or those without clinical and demographic details were excluded.

### Data extraction

The genotype, phenotype, and country of origin of published WRS cases were retrieved from the full text of the articles. The following data were collected and described: mutation type, gender, gestational age, birth weight, age at diagnosis, age at reporting or death, clinical phenotype, and prognosis. The total number of families and subjects with WRS from KSA was compared to published cases from other countries and to the total number of reported patients with PNDM from KSA.

## Results

**Fig. 1:**
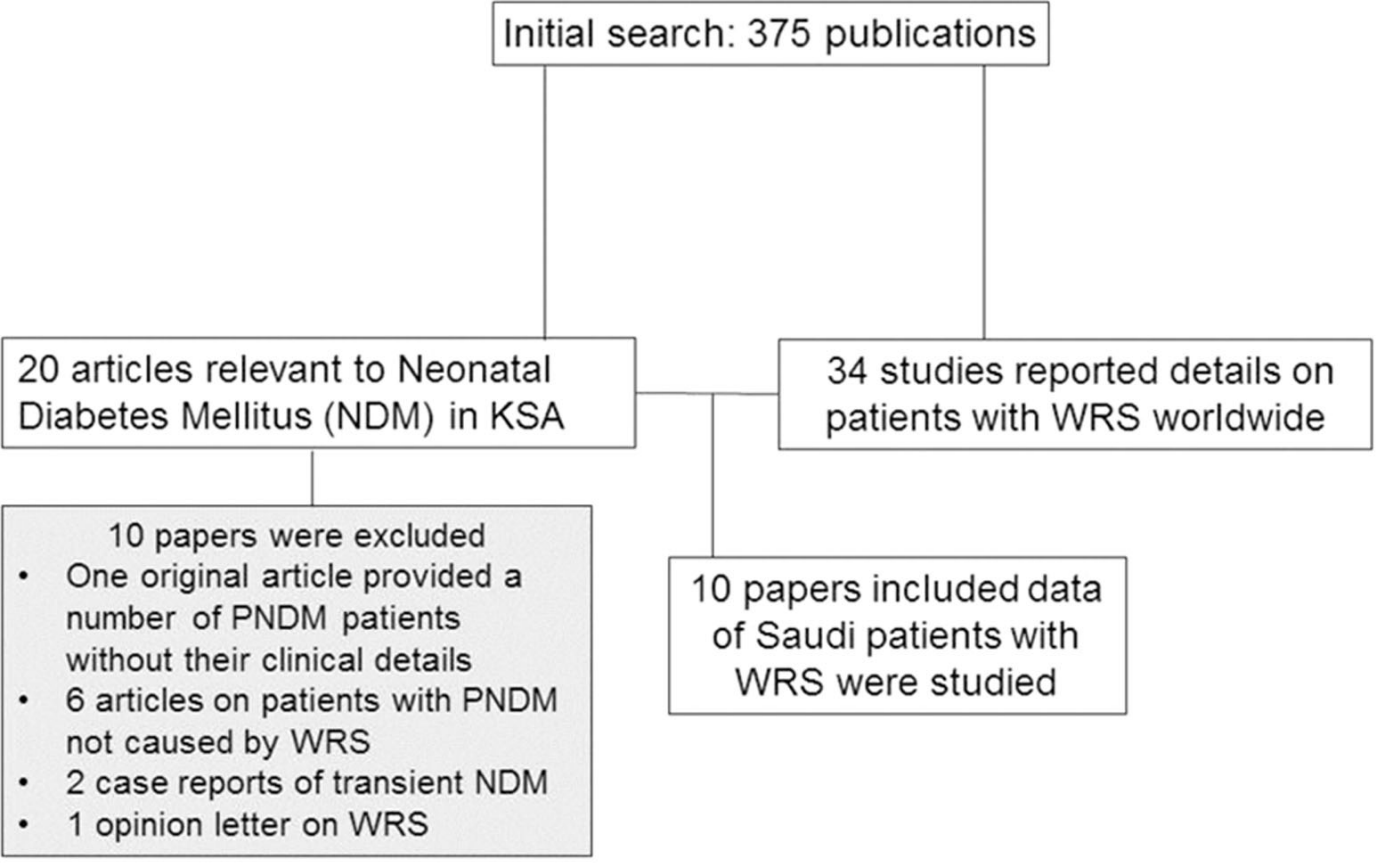
Details of data selection.

The data selection process is shown in Fig. 1. Ten articles (five original reports) published between 1995 and 2012 met the inclusion and exclusion criteria. Of the original articles, two papers reported Saudi patients as part of an international WRS cohort (3, 4), one provided data on the frequency and spectrum of WRS in one KSA region (14), and two described studies to define the genetic causes of Saudi subjects with a clinical phenotype of WRS (15, 16). The five case reports (17–21) were on patients; most of them had been or were subsequently duplicated elsewhere (Table 1). Seven of the published Saudi patients with WRS were reported in more than one article (Table 1).

**Table 1:** Summary of demography, mutations, and phenotype of WRS families reported from KSA until 31 January 2013

Family and patient number	References	EIF2AK3 mutation: protein (nucleotide)	Sex	Birth weight (kg)	DM onset (weeks)	Skeletal dysplasia	Recurrent hepatitis	Other reported features and prognosis
1.1	(14)	V349SfsX3 (c.1044_1057del)	F	2.4	10	Yes	Yes	Central hypothyroidism; died at 2 yr
1.2	(4, 14)	V349SfsX3 (c.1044_1057del)	F	2.9	8	No	Yes	None; alive on insulin
1.3	(14)	V349SfsX3 (c.1044_1057del)	M	3.1	6	No	Yes	None; died at 2 yr with acute hepatitis
2.1	(14)	V349SfsX3 (c.1044_1057del)	F	2.0	16	Yes	Yes	Developmental delay, short stature, and renal failure; died at 6 yr
2.2	(14)	V349SfsX3 (c.1044_1057del)	M	3.1	21	Yes	Yes	Short stature, renal dysfunction; died at 3 yr
3.1	(14)	V349SfsX3 (c.1044_1057del)	M	2.34	8	No	Yes	No other features; died at 6 yr
3.2	(14)	V349SfsX3 (c.1044_1057del)	M	2.40	8	No	Yes	No other features; alive on insulin
4.1	(4, 17)	N420TfsX14 (c.1259delA	M	2.75	3	Yes	Yes	Short stature; died at 7 yr
4.2	(17)	N420TfsX14 (c.1259delA	F	3.2	19	No	Yes	Transient hypothyroidism; alive at 1.1 yr
4.3	(17)	N420TfsX14 (c.1259delA	M	3.1	4	No	Yes	Died at 1.1 yr with acute hepatitis
5.1	(17)	N420TfsX14 (c.1259delA	F	2.4	12	No	No	Transient hypothyroidism; alive at 3.5 yr
5.2	(17)	N420TfsX14 (c.1259delA	F	2.7	8	Yes	Yes	Ectopic left kidney; depressed nasal; bridge; short neck, upward slanting of palpebral fissures; WPW syndrome; alive at 2 yr on insulin
6.1	(4)	S469X(c.1406C>G)	F	2.0	4	Yes	Yes	Primary hypothyroidism at 1.3 yr (TSH:146.6 mU/L, FT4<5 pmol/L), hepatomegaly; alive at 2 yr
7.1	(4)	R1064X (c.3190C>T)	M	2.8	20	Yes	Yes	Congenital nystagmus, microcephaly, neutropenia, and anemia at 1 yr; alive at 4 yr
8.1	(4)	A159PfsX41 (c.475delG)	M	3.3	6	Yes	Yes	Neutropenia; alive at 3 yr
9.1	(15, 16, 18)	E524X (1563delGAAA)	M	1.1 at 28 wks	1	Yes	No	Proptosis, high arched palate, downward slanting of palpebral fissure, hydrocephalus, blue sclera, neurodevelopmental delay, recurrent hypoglycemia, osteopenia, and spontaneous fracture; died at 2 yr.
9.2	(15)	Not tested	M	1.5 at 30 wks	4	No	No	Alive at 5 months
10.1	(16, 18)	IVS14+1G/A (sp996X1020	M	2.5	8	Yes	No	Severe neurodevelopmental delay, recurrent hypoglycemia; died of renal failure at 4 yr
10.2	(16)	Not tested	M	NR	2	No	No	Alive at 9 yr
11.1	(3, 19, 20)	W164X	M	2.2	6	Yes	Yes	Hepatomegaly, developmental delay, renal dysfunction; alive at 6 yr
11.2	(3)	W164X	F	NR	8	NR	NR	Patient was too young at the time of reporting to show other features; alive
12.1	(3, 21)	Fs1025X1048	F	1.7	8	Yes	Yes	Central hypothyroidism, hepatomegaly, depressed nasal bridge, developmental delay; alive at 6 yr
12.2	(3, 21)	Fs1025X1048	M	1.1	8	Yes	Yes	Central hypothyroidism, mild pancreatic hypoplasia on CT; died at 5 yr

NR, not reported.

### Frequency of WRS

**Fig. 2:**
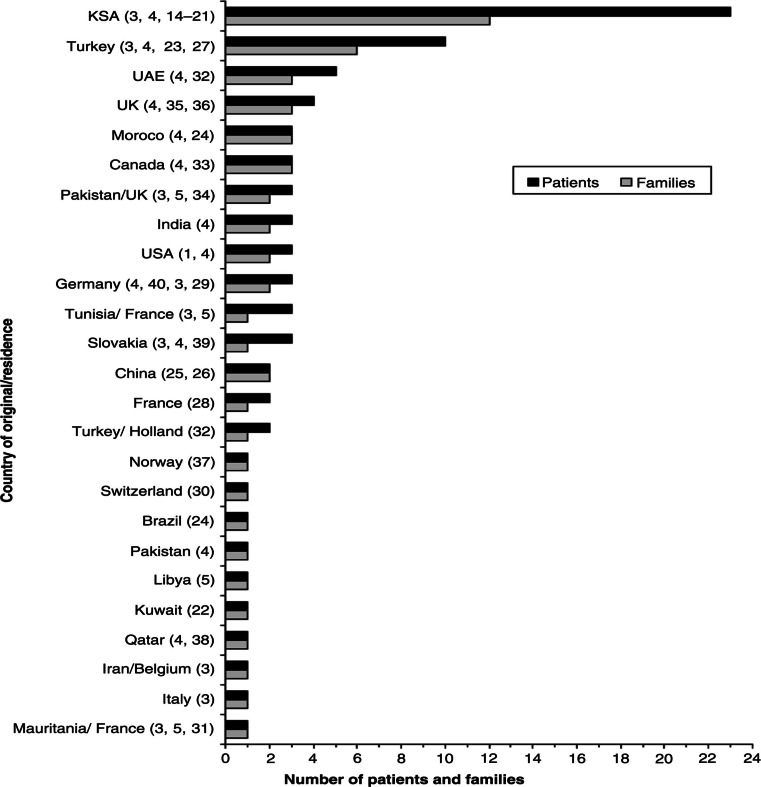
The number of patients and families with WRS per country of origin or residency reported until January 2013. UAE=United Arab Emirates; UK=United Kingdom; USA=United States of America. The numbers between brackets indicate the references.

In total, 23 children (14 boys) with WRS from 12 Saudi families were identified (3, 4, 14–21). This number accounts for 27.7% (23/83) of patients and 22.2% (12/54) of families with WRS reported worldwide until 31 January 2013 (1, 3–5, 14–40). A comparison between the number of patients and families with WRS from KSA and those from other countries is illustrated in Fig. 2. The 23 children with WRS represent 58.9% of the 39 patients with PNDM reported from KSA to date (3, 4, 14–21, 41–46).

### Clinical phenotype

The clinical characteristics of the 23 Saudi patients are summarized in Table 1. All children except one (8.1) were the product of consanguineous marriages, and 60% of them were males. Two siblings (9.1 and 9.2) were born preterm and the rest were born at term, with a mean birth weight of 2.52 kg. PNDM was the presenting feature in all patients with a median age of onset at 15 weeks. There was a variation in the frequency of other features even between affected siblings of the same gender (Table 1). At the time of reporting, 39.1% (9/23) of the patients were dead, and the longest surviving patient was 9 years old. Recurrent hepatitis was experienced in 73.9% (17/23) of patients, and skeletal dysplasia was diagnosed in 56.5% (13/23) of them. Thyroid dysfunction was documented in 26% (6/23) of patients: one patient developed primary hypothyroidism in the first year of life, and the other five had picture suggestive of central hypothyroidism. Impaired renal function was reported in only three subjects, and neutropenia was observed in two patients from different families. Three children were reported to have growth failure; however, exocrine pancreatic function was not tested in any of them.

### Genotype

Genetic diagnosis of WRS was confirmed in all 12 Saudi families (Table 1), and their affected members were homozygous for nine different *EIF2AK3* mutations spreading through the PERK protein and leading to truncated proteins (4 nonsense, 4 frameshift, and one deletion at exon 14/intron boundaries of the *EIF2AK3* gene). Of these mutations, eight were confined to these families, and one nonsense mutation (E523X) was also reported in a child from Qatar (4). Three families from the same tribe shared the same frameshift V349SfsX3 mutation, and two unrelated families have an identical frameshift N420TfsX14 mutation. No missense mutations were identified, and there was no correlation between the genotype and phenotype.

## Discussion

This article provides comparative data on the frequency of WRS between different countries and reviews the genotype and phenotype of WRS patients reported from KSA.

Since the latest literature review on WRS by Julier and Nicolino in 2010 (2), 8 families (14 patients) from five countries have been reported (14, 17, 23–26), making a total number of 54 families (83 patients) reported with this condition worldwide until January 2013. The published data show a variation in the number of reported families between different countries, with more cases from populations where consanguinity is part of the culture such as the Arab states and Turkey (Fig. 1). This variation may be related to different factors, such as the population size, consanguinity rate, and allele frequency. It could also reflect the level of recognition of WRS and publication interest among clinicians between different countries.

Strikingly, 22.2% of these families were from KSA, which has the largest collection of patients with WRS reported for a single country, suggesting that KSA is a hotspot for this condition. The most likely explanation for the higher frequency of WRS in KSA compared to other countries is the combination of a large population and a high consanguinity rate of 56% (13). All patients except one were the product of consanguineous marriages, and the condition is the commonest cause of PNDM in consanguineous families (4). The 23 WRS patients represent almost 60% of the reported patients with PNDM from KSA (2, 3, 14–21, 41–46). This finding is in line with recent reports that showed that WRS is the commonest cause of PNDM in northwest KSA (14) and in the Arab population (12). It is expected that not all cases with WRS in KSA were reported, and it is possible that some patients with this condition deceased before the diagnosis was made. Given the high birth rate and consanguinity in KSA, the author suspects that the reported number of WRS cases is probably underestimating the frequency of this condition in the country. There is no doubt that launching a national survey and establishing a registry for NDM and WRS in KSA would raise the awareness of the condition and provide more accurate data on the epidemiology of WRS and other forms of NDM.

All WRS patients from KSA presented with PNDM; however, there was a variation in the onset, nature, and severity of other features even between affected siblings of the same gender (Table 1). This phenomenon has been reported in some WRS families from other countries (3, 4) and has been attributed to various factors, such as the presence of other modified genes, variable expression of the *EIF2AK3* gene, epigenetic factors, and variation in management protocols. However, it is also possible that some of these patients were still too young to show some features, such as skeletal dysplasia and failure to thrive, at the time of reporting, or they were not completely assessed for less common features of WRS. Of note, neurodevelopmental functions were not formally assessed in most Saudi subjects, and none of them had their stool elastase measured to diagnose exocrine pancreatic dysfunction.

Around 40% of children from KSA were dead at the time of reporting compared to 70% in the 10 reported patients from Turkey (3, 4, 23, 27), which has the second largest number of reported cases. Recurrent hepatitis triggered by viral infection was reported in more than 70% of Saudi patients, and acute fulminant hepatitis was the main cause of death in all deceased patients from KSA. Recent attempts to reduce ER stress through different mechanisms are promising (47, 48); however, it is important to ensure that families of affected children recognize early symptoms of liver dysfunction and that patients with WRS have a clear plan prepared to avoid delay in starting the management of acute hepatic failure. Liver transplantation was successfully used in one patient with acute fulminant hepatitis and PNDM from the United Arab Emirates (UAE) in whom the diagnosis of WRS was later confirmed at the molecular level (4). Interestingly, his long-term diabetes control has improved following the transplantation (A. Deeb, personal communication). However, no patient from KSA was offered this therapy, possibly due to the short life expectancy of these children and the lack of international experience with liver transplantation in the condition. Given the high mortality and morbidity of the condition, the author suggests that patients with WRS should be managed by a multidisciplinary team (consisting of an endocrinologist, a hepatologist, a geneticist, and orthopedic surgeons) and believes that developing guidelines through a national consortium would improve the prognosis of these children.

In the reported Saudi children with WRS, five have thyroid function consistent with central hypothyroidism (Table 1). However, in two patients, the changes were transient in keeping with euthyroid sickness (ES), while in the remaining three patients the thyroid function tests were not repeated. The author supports the notion by Ozbek et al. (27) and Al-Shawi et al. (17) that thyroid dysfunction is a reflection of ES rather than a feature of WRS. Two patients from separate families (5.2 and 9.1) were described as having facial dysmorphism, which has been reported only in WRS cases from KSA; however, it was not clear whether chromosomal karyotyping was performed to exclude other conditions associated with these features. A long-term follow-up study would provide more insight into the clinical phenotype and prognosis of WRS.

The molecular diagnosis of WRS was confirmed in all reported cases from KSA, with no correlation between the phenotype and genotype in the 12 Saudi families as reported in most families with WRS (3, 4, 27). Three families from the same tribe shared the same frameshift mutation (V349SfsX3), and two unrelated families have the same N420TfsX mutation, indicating a founder effect or mutation hotspot. Eight of the nine *EIF2AK3* mutations were identified in only these Saudi families; however, one mutation (E523) was also reported in a child from Qatar (4, 38). The fact that Qatar is a neighboring Arab state raises the possibility of a founder effect. It appears that these nine mutations are confined to Arab populations; however, a large study would confirm this possibility.

Of the *EIF2AK3* mutations reported to date, four appeared to be associated with a specific phenotype: a delayed age of onset for diabetes was reported with the N656 and I650 mutations (3, 4, 31), whereas milder courses with prolonged survivals of 32 years and 35 years were reported with the F593 and L646 mutations, respectively (3, 4, 29). Interestingly, all of them were missense mutations located closer to each other on the first kinase domain of PERK protein residue. However, no missense mutations were identified in the Saudi families, which could explain the early onset of diabetes and shorter survival age in all KSA patients. Neutropenia is another feature thought to be associated with certain mutations (2, 3, 27). However, it was reported in only two of three affected males from a UAE family with the W430X mutation, and it was transient in a single affected family member with the K150RfsX2 mutation in the Rubio-Cabizas series (4). Out of the nine reported mutations from KSA, neutropenia was reported with only the R1064X and A159PfsX41 mutations; however, these mutations are not close to each other on the PERK protein, and each family has only one affected boy. It is therefore difficult to conclude whether certain *EIF2AK3* mutations are associated with neutropenia, at least in Saudi families with WRS.

The diagnosis of WRS is usually made on the combination of the typical phenotype and confirmed by genetic testing. However, Rubio-Cabizas et al. reported three probands with apparent isolated PNDM in whom the molecular diagnosis was made before the appearance of other clinical features. Of note, one of the Saudi patients (2.1) has isolated PNDM, and the diagnosis of WRS was made only by genetic testing at 3.5 year olds following the presentation of her younger sister (17). Given the high morbidity, high mortality, and frequency of WRS in KSA, facilities for early genetic testing should be made readily available for all patients with PNDM, and all patients with PNDM from KSA and from other states where consanguinity is highly practiced should be tested for *EIF2AK3* mutations regardless of their phenotype. Reducing consanguinity is a challenge in some cultures such as KSA; however, antenatal diagnosis with termination of pregnancy and pregestational diagnosis, which allow for the selection of unaffected zygotes, can be offered, particularly for families with more than one affected child.

## Conclusions

Data on the frequency of WRS are sparse, and information on this condition in KSA is limited to a few case reports or as part of international series. However, the available data show that KSA has the highest reported cases of WRS worldwide, and the condition is the commonest cause of PNDM in the country. A national and international registry of WRS and PNDM based on regular surveys would raise awareness of the condition, provide accurate data, and help in clinical management. It is also important that facilities for genetic testing for WRS should be available for clinicians in order to confirm the diagnosis and provide families with proper genetic counseling.
